# Developing psychiatric resources for the new foundation programme curriculum

**DOI:** 10.1192/bjo.2021.408

**Published:** 2021-06-18

**Authors:** Drew Kinmond, Fiona Hynes, Aqib (Mohammad) Hussain

**Affiliations:** 1 Reaside Clinic, Birmingham and Solihull Mental Health Foundation Trust; 2 Birmingham Sollihull Mental health foundation trust

## Abstract

**Aims:**

Our aim was to develop an easily accessible, relevant and deliverable resource to meet the training requirements of the new foundation curriculum for Foundation Trainees in the West Midlands. The virtual resource needed to provide information at the correct knowledge depth, whilst also being flexible enough to allow trainees to access the materials despite the challenges of remote working. The West Midlands currently holds approximately 1,300 places for foundation training with an increase in numbers planned for 2023 and 2024.

**Method:**

The United Kingdom Foundation Programme (FP) is a two-year structured, supervised training programme of learning in the workplace developed to prepare medical graduates for speciality training. The Foundation Curriculum is currently being updated in line with the GMC Standards for Post Graduate Curricula to reflect the developing and contemporaneous training needs of doctors and is expected to go live in August 2021.

Though the foundation curriculum is broad and does not usually include specific diseases, it is recognised that mental health disorders are common and are frequently missed. The new curriculum makes a specific statement regarding the importance of mental health and specifies a syllabus covering this important area of medical practice

Around 80% of doctors are expected to have exposure to a community medicine placement, with around 40% expected to have placement within a specific mental health setting. Though other community placements may provide some exposure to the acute challenges of mental health, this is not guaranteed.

To assist in meeting the FP requirement for training in mental health we developed an online resource for all West Midlands trainees, with lectures and information available that covers all of the core curriculum requirements for the FP. These resources can be accessed at any time of the day, at any point of foundation training, with each module certificated to show evidence of the attainment of foundation competencies ready for students ARCP (Annual Review of Competency Progression).

**Result:**

A programme of evaluation and effectiveness will be undertaken when the new curriculum goes live.

**Conclusion:**

There is an expected expansion in the number of training Foundation doctors within the next 5 years; therefore the demand for this training is expected to increase over time. As the understanding and awareness of the interaction between physical health and mental health continues to develop, we expected the use of this resource grow into the future.

